# Antibody-mediated depletion of programmed death 1-positive (PD-1^+^) cells

**DOI:** 10.1016/j.jconrel.2022.07.010

**Published:** 2022-07-15

**Authors:** Yujia Zhai, Shuyun Dong, Haojia Li, Yue Zhang, Paul Shami, Mingnan Chen

**Affiliations:** a Department of Pharmaceutics and Pharmaceutical Chemistry, University of Utah, Salt Lake City, UT 84112, USA; b Division of Epidemiology, Department of Internal Medicine, University of Utah, Salt Lake City, UT 84132, USA; c Division of Hematology and Hematologic Malignancies, Department of Internal Medicine, and Huntsman Cancer Institute, University of Utah, Salt Lake City, UT 84112, USA

**Keywords:** Programmed death-1, PD-1, PD-1-positive cell, PD-1^+^ cell, Depleting antibody, Antibody-mediated cell phagocytosis, Complement-dependent cytotoxicity cancer, Autoimmune diseases

## Abstract

PD-1 immune checkpoint has been intensively investigated in pathogenesis and treatments for cancer and autoimmune diseases. Cells that express PD-1 (PD-1^+^ cells) draw ever-increasing attention in cancer and autoimmune disease research although the role of PD-1^+^ cells in the progression and treatments of these diseases remains largely ambiguous. One definite approach to elucidate their roles is to deplete these cells in disease settings and examine how the depletion impacts disease progression and treatments. To execute the depletion, we designed and generated the first depleting antibody (D-αPD-1) that specifically ablates PD-1^+^ cells. D-αPD-1 has the same variable domains as an anti-mouse PD-1 blocking antibody (RMP1–14). The constant domains of D-αPD-1 were derived from mouse IgG2a heavy and κ-light chain, respectively. D-αPD-1 was verified to bind with mouse PD-1 as well as mouse FcγRIV, an immuno-activating Fc receptor. The cell depletion effect of D-αPD-1 was confirmed *in vivo* using a PD-1^+^ cell transferring model. Since transferred PD-1^+^ cells, EL4 cells, are tumorigenic and EL4 tumors are lethal to host mice, the depleting effect of D-αPD-1 was also manifested by an absolute survival among the antibody-treated mice while groups receiving control treatments had median survival time of merely approximately 30 days. Furthermore, we found that D-αPD-1 leads to elimination of PD-1^+^ cells through antibody-dependent cell-mediate phagocytosis (ADCP) and complement-dependent cytotoxicity (CDC) mechanisms. These results, altogether, confirmed the specificity and effectiveness of D-αPD-1. The results also highlighted that D-αPD-1 is a robust tool to study PD-1+ cells in cancer and autoimmune diseases and a potential therapeutic for these diseases.

## 1. Introduction

PD-1 immune checkpoint is one critical mechanism to maintain immunostasis. The checkpoint counteracts immune stimulatory signals and hence, negatively regulates immune responses. PD-1 immune checkpoint is triggered when PD-1, a receptor, engages with its ligands, PD-L1 or PD-L2. PD-1 was first found to be expressed on activated B and T cells when they differentiate into effector cells [1–3]. Recently, PD-1 was also found to be expressed on other immune cells including activated NK cells, macrophages, and innate lymphoid cells [4,5]. PD-L1 and PD-L2 are found to express on a wide range of cells including dendritic cells, macrophages, B cells, and some non-immune peripheral cells [6]. To date, the PD-1 immune checkpoint has been connected with pathogenesis and treatments for several types of disorders including autoimmune diseases, cancer, and chronic infections [5,7,8,10]. For example, the knockout of PD-1 in NOD mice exacerbates type-1 diabetes in mice; the knockout in C57B/L6 mice confer a lupus-like phenotype in these mice [11]. The broad implication of PD-1 immune checkpoint in various diseases also brought attention to cells that express PD-1, generally termed as PD-1-positive (PD-1^+^) cells. PD-1^+^ cells encompass different populations of cells and play drastically different roles in different disease settings.

In the context of autoimmune disease, PD-1^+^ cells refer to effector T and B cells that exert autoimmune attacks although PD-1^+^ Tregs may also influence the initiation and progression of certain diseases. Tissue infiltration of PD-1^+^ cells was found to increase the progression of autoimmune diseases [8]. Another fact is that PD-1^+^ cell proliferation worsens the condition of mice and patients with autoimmune disease [8,12–14]. Interestingly, the PD-1 immune checkpoint intrinsically functions to suppress autoimmunity. The observation that autoimmune diseases start and progress despite the normality of the PD-1 immune checkpoint suggests the immune checkpoint may be overridden by other autoimmunity-driving factors [5,7]. Clinically and preclinically, it has been reported that a blockade of the PD-1 immune checkpoint, a treatment that boosts PD-1^+^ lymphocytes, exacerbate autoimmune disorders [8,12–14]. Based on these observations, PD-1^+^ cells may be largely viewed as pathogenic cells of autoimmune disease. Consequently, the suppression and depletion of PD-1^+^ cells may be an effective strategy to ameliorate autoimmune disease. Our recent research has proven therapeutic effects of the depletion of PD-1^+^ cells in mouse models of experimental autoimmune encephalomyelitis (EAE) and Type-1 diabetes [15]. These results show the promise to exploit PD-1^+^ cells as a therapeutic target of autoimmune treatments.

PD-1^+^ cells are also highlighted in cancer immunotherapy. PD-1^+^ T cells are believed to be major effector cells in the success of PD-1 immune checkpoint therapy [16,17]. However, PD-1^+^ Tregs and PD-1^+^ cancer cells are also worthy of attention when investigating factors that cause resistance to PD-1 immune checkpoint therapy; the resistance is indeed common among patients who receive the therapy [18–23]. Tumor cells that intrinsically express PD-1 were recently discovered in many types of tumors [19,24–28]. These cancer cells were proposed to play roles in tumorigenesis and the resistance to the PD-1 immune checkpoint therapy [24–27,29,30]. However, apparently competing views exist regarding the relationship between these cells and the immune checkpoint blockade. According to one side of the view, PD-1 on tumor cell functions as a tumor suppressor, and theoretically, a blockade of the PD-1 results in a promotion of tumor growth [24]. The other side of the view, however, believes that tumor cell-intrinsic PD-1 acts to facilitate tumor growth [25] and that PD-1^+^ tumor cells are tumor initiating cells [28]. Such contradictory views may be partially attributed to the research approaches through which these conclusions were reached: the PD-1 blockade, PD-1 over-expression, and PD-1 knockout [24,25,27,29]. These approaches would not be able to reveal the impacts of PD-1^+^ tumor cells as a whole, specifically regarding the tumorigenesis and resistance to the PD-1 immune checkpoint therapy. As such, the depletion of PD-1^+^ tumor cells may reveal some novel insights on the impacts of the PD-1 immune checkpoint therapy and help to clarify the aforementioned controversy.

Previously, we generated an immunotoxin that specifically targeted PD-1^+^ cells [15]. For mice with EAE and type-1 diabetes, the treatment of the PD-1 immunotoxin reduced PD-1+ lymphocytes in these mice and ameliorated their autoimmune diseases. On the other hand, the administration of the PD-1 immunotoxin did not compromise healthy adaptive immunity, evidenced by full-strength immune responses to vaccinations in treated mice to vaccinations. Since depletion of PD-1^+^ cells has shown its potential as a treatment for a wide range of autoimmune diseases and is promised to serve as a method or a therapeutic strategy for cancer, it is worthwhile to diversify tools to execute depletion. Depleting antibodies represent a promising direction. Depleting antibodies have been used clinically for a wide range of diseases with proven safety, scalability, and industrial feasibility. Another advantage of antibodies is their long plasma half-lives (10–21 days in human) due to their high affinity binding with FcRn and subsequent escape of endosomal degradation [31]. Depleting antibodies may cause the elimination of the cells that express corresponding antigens by Fc-mediated effector mechanisms including antibody-dependent cell-mediated cytotoxicity (ADCC), complement dependent cytotoxicity (CDC) and antibody-dependent cell-mediated phagocytosis (ADCP). CDC and ADCP relies on the binding between depleting antibodies and FcγR on effector cells such as macrophages [32]. Mouse FcγRIV is one type of activation FcR expressed on macrophages, monocytes, and neutrophils [33] and has been chosen as a target during the development of mouse depleting antibodies [34]. Among all mouse IgG subtypes, IgG2a and IgG2b antibodies show the highest binding affinity with the FcγRIV [33]. Different from IgG2b ones, mouse IgG2a antibodies also has a high binding affinity with mouse FcγRI, which expresses on monocytic dendritic cells and contributes to depleting effect of the antibodies [35]. Additionally, mouse IgG2a antibodies have long plasma half-lives as of 5–8 days [36–39]. Thus, it is desirable to engineer mouse depleting antibodies of the IgG2a subtype.

Here, we engineered and generated the very first antibody aimed at depleting PD-1^+^ cells, D-αPD-1. D-αPD-1 consists of variable domains of a known anti-mouse PD-1 antibody (clone RMP1–14, termed B-αPD-1 hereafter) and constant domains from mouse IgG2a heavy chain and κ-light chain. D-αPD-1 is able to bind specifically to PD-1^+^ cells. Importantly, D-αPD-1 is able to eliminate PD-1^+^ cells *in vivo*. Last, D-αPD-1 was found to utilize CDC and ADCP to abolish PD-1^+^ cells. In summary, we provided an effective tool to deplete PD-1^+^ cells through which we may investigate the role and function of PD-1^+^ cells in cancer and autoimmune diseases, among others.

## 2. Results

### 2.1. Design and Generation of a mouse depleting anti-PD-1 antibody (D-αPD-1)

**Fig. 1.**
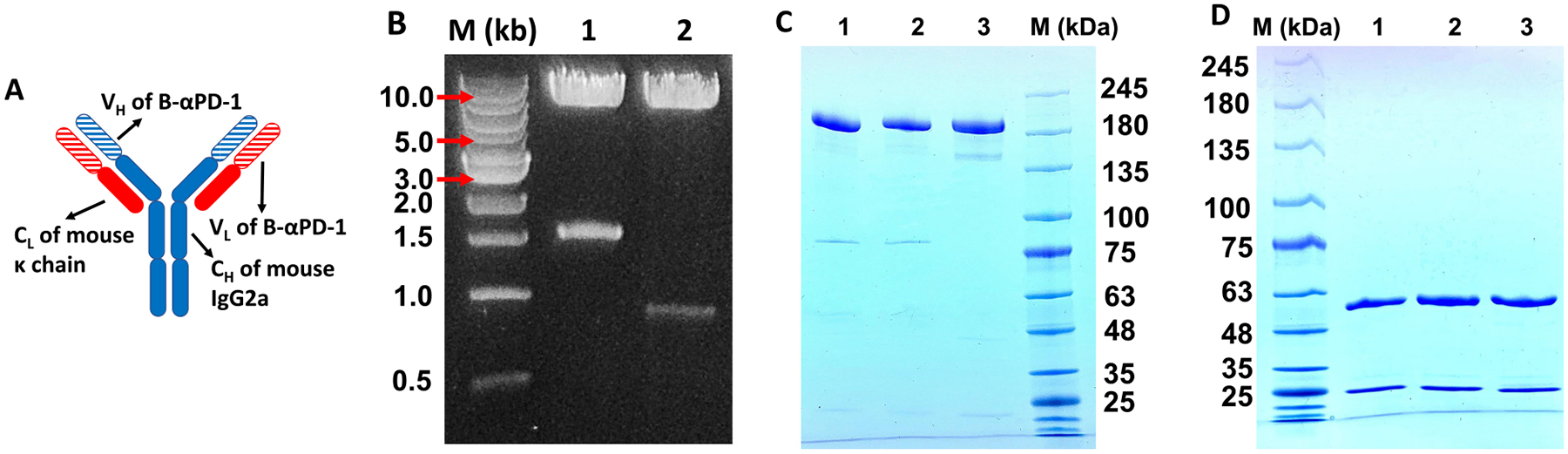
The design and generation of D-αPD-1. (A) The schematic showing the design of D-αPD-1. Blue fragments are expressed by the heavy chain plasmid, and red fragments are expressed by the light chain plasmid. (B) The agarose gel image of plasmid digestion products from the plasmid encoding heavy chain (lane 1) and the plasmid encoding light chain (lane 2). Heavy chain plasmid DNA was digested using *Xba*I and *Eco*RV. Light chain plasmid DNA was digested using *Sac*I and EcoRV. The upper bands of each lane represent cleaved vectors; the lower bands of each lane represent the coding genes of heavy chain and light chain respectively. (C) The non-reducing SDS-PAGE image of D-αPD-1 (lane 1) after purification, compared with B-αPD-1 (lane 2) and commercial IgG2a (lane 3). (D) The reducing SDS-PAGE gel of D-αPD-1 (lane 1) compared with B-αPD-1 (lane 2) and commercial IgG2a (lane3). The upper band represents the heavy chain; the lower band shows the light chain.

D-αPD-1 was produced as two recombinant proteins, a heavy chain and a light chain through a mammalian expression system. Then, the heavy chain and the light chain self-assembled into D-αPD-1 inside mammalian host cells. The coding gene for D-αPD-1 heavy chain was generated by fusing two genes together: one gene is for the heavy chain variable domain (V_H_) of B-αPD-1 (RMP1–14) [15]; another gene is for three heavy chain constant domains of a mouse IgG2a (Genbank ID: BAC44883.1). The coding gene of D-αPD-1 light chain was generated by fusing two genes as well: one gene is for the light chain variable domain (V_L_) of B-αPD-1; another gene is for the constant domain of a mouse κ chain (GenBank: BAB33404.1) (Fig. 1A). The coding genes of D-αPD-1 heavy and light chains were chemically synthesized and inserted in a pcDNA3.4 expression vector, respectively (Fig. 1A). The sizes of inserted coding genes were examined through enzymatic cleavage in combination with gel electrophoresis. The migration positions of inserted genes for D-αPD-1 light and heavy chains on the gel suggest that the sizes of these two genes are approximately 700 bp and 1500 bp, respectively. These estimated sizes are consistent with the theoretical sizes of the coding genes (light chain 723 bp and heavy chain 1413 bp, (Fig. 1B)). The sequence of coding genes on the expression vectors were confirmed by DNA sequencing.

To express D-αPD-1, the expression vectors harboring encoding genes of light and heavy chains were used to co-transfect Expi293F cells at the 2:1 ratio as suggested by the protocol of the expression system. The assembled D-αPD-1 was secreted from Expi293F cells. D-αPD-1 was purified using protein G beads. On a non-reducing SDS-PAGE gel, D-αPD-1 appear to have a similar MW to B-αPD-1 and an IgG2a isotype control. On a reduced SDS gel, D-αPD-1 shows in two bands: a higher band of approximately 50 kDa represents heavy chains and a lower band of approximately 25 kDa represents light chains (Fig. 1D).

### 2.2. D-αPD-1 selectively binds to PD-1+ cells

**Fig. 2.**
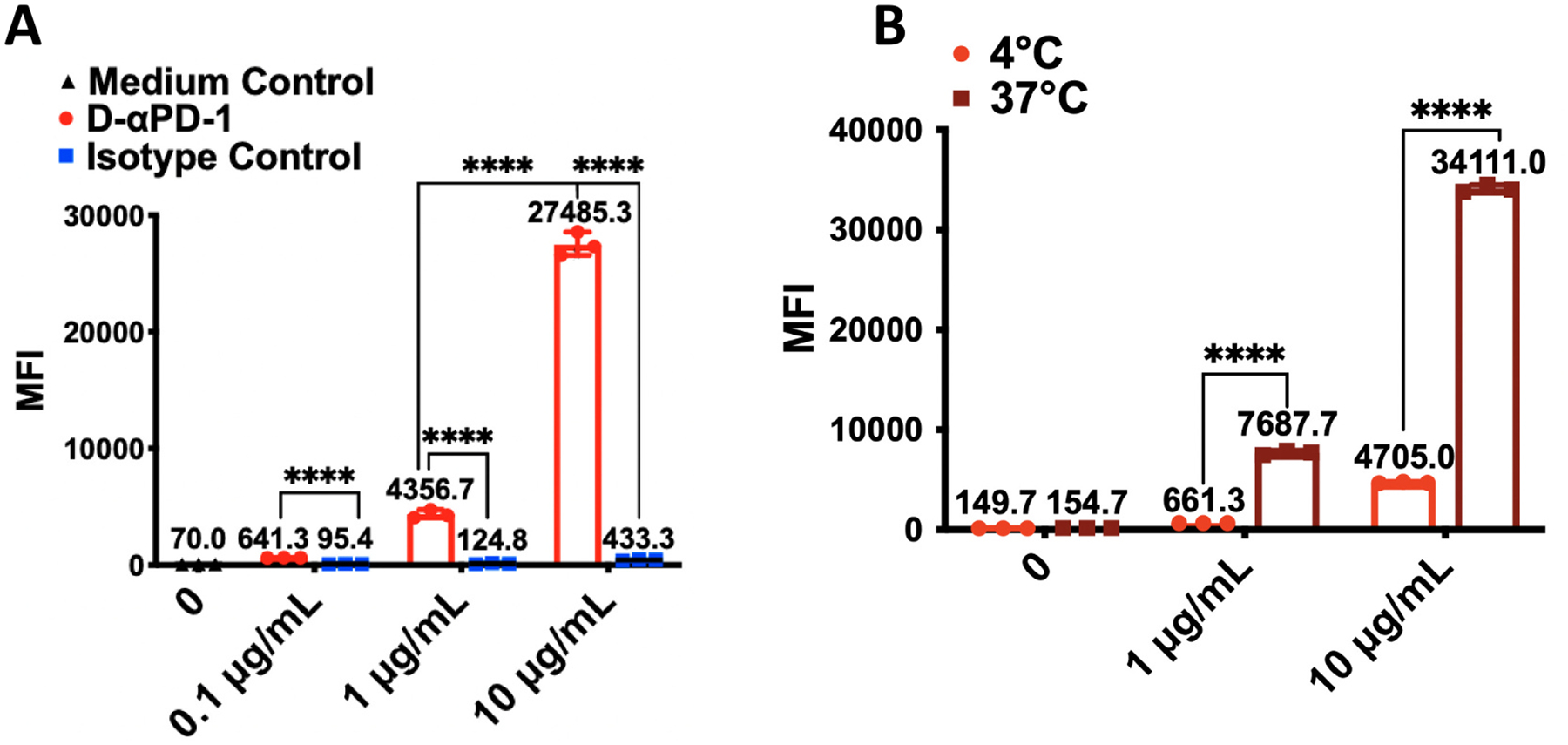
Interactions between D-αPD-1 and EL4 cells. (A) The MFI of EL4 cells after the cells were incubated with D-αPD-1 or mouse IgG2a at different concentrations on ice for 30 min, then were stained with PE-anti-mouse-IgG2a Ab and analyzed by flow cytometry. (B) The MFI of EL4 cells after the cells were incubated with Alexa-647-labeled D-αPD-1 at different concentrations at 4 °C or 37 °C for 1 h. The data are presented as means ± SD of each treatment (*N* = 3). (****P < 0.0001).

We first studied interactions between D-αPD-1 and cells at 4 °C, a condition that allows for the binding between an antibody and its cell surface antigens but inhibits the internalization of the antigens. Here, D-αPD-1 and an IgG2a isotype control at three different concentrations were incubated with EL4 cells that are PD-1^+^. Then, bound antibodies were stained with PE-labeled, anti-mouse IgG2a antibody and measured by flow cytometry. The mean MFI resulted from the incubation between EL4 cells and the isotype control is statistically significantly lower than that from the incubation between EL4 cells and D-αPD-1 at all three concentration settings (*P* < 0.0001 Fig. 2A). Further, the binding between D-αPD-1 and EL4 cells was found to be dose-dependent (Fig. 2A). For the three used concentrations, 0.1, 1.0, 10.0 μg/mL, the higher the D-αPD-1 concentration, the greater the mean MFI. Specifically, when the concentration of D-αPD-1 increased from 1 μg/mL to 10 μg/mL, the mean of MFI raised by approximately 6 times (4356.7 *vs* 27,485.3, P < 0.0001). In contrast, the increased concentrations of the isotope control did not result in an increase of MFI. All together, these results suggested that D-αPD-1 is able to bind with PD-1^+^ cells, which is the basis for the depletive effect of D-αPD-1.

Next, we compared interactions between D-αPD-1 and cells at 4 °C and 37 °C. The latter condition allows not only the binding of antibodies with their cell surface antigens but also the internalization of the antigens. To conduct the comparison, we incubated Alexa Fluor 647-labeled D-αPD-1 with EL4 cells at two different concentrations (1 and 10 μg/mL) and two different temperatures. After that, the cells were analyzed by flow cytometry. When no D-αPD-1 is used for incubation, EL4 cells have low and similar MFIs at both temperatures. When 1 μg/mL D-αPD-1 was used for the incubation, the mean MFL at 37 °C is approximately 10 times higher than that at 4 °C (7687.7 *vs* 661.3, *P* < 0.0001). This trend is maintained when D-αPD-1 was used at 10 μg/mL. The mean MFI resulted from the 37 °C incubations is >7-time greater than the mean MFI from the 4 °C incubations (34,111.0 *vs* 4705.0, P < 0.0001). These data clearly showed that at 37 °C, a greater amount of D-αPD-1 is associated with EL4 cells, which may be caused by internalization of PD-1.

### 2.3. The treatment of D-αPD-1 leads to elimination of PD-1^+^ cells in vivo

**Fig. 3.**
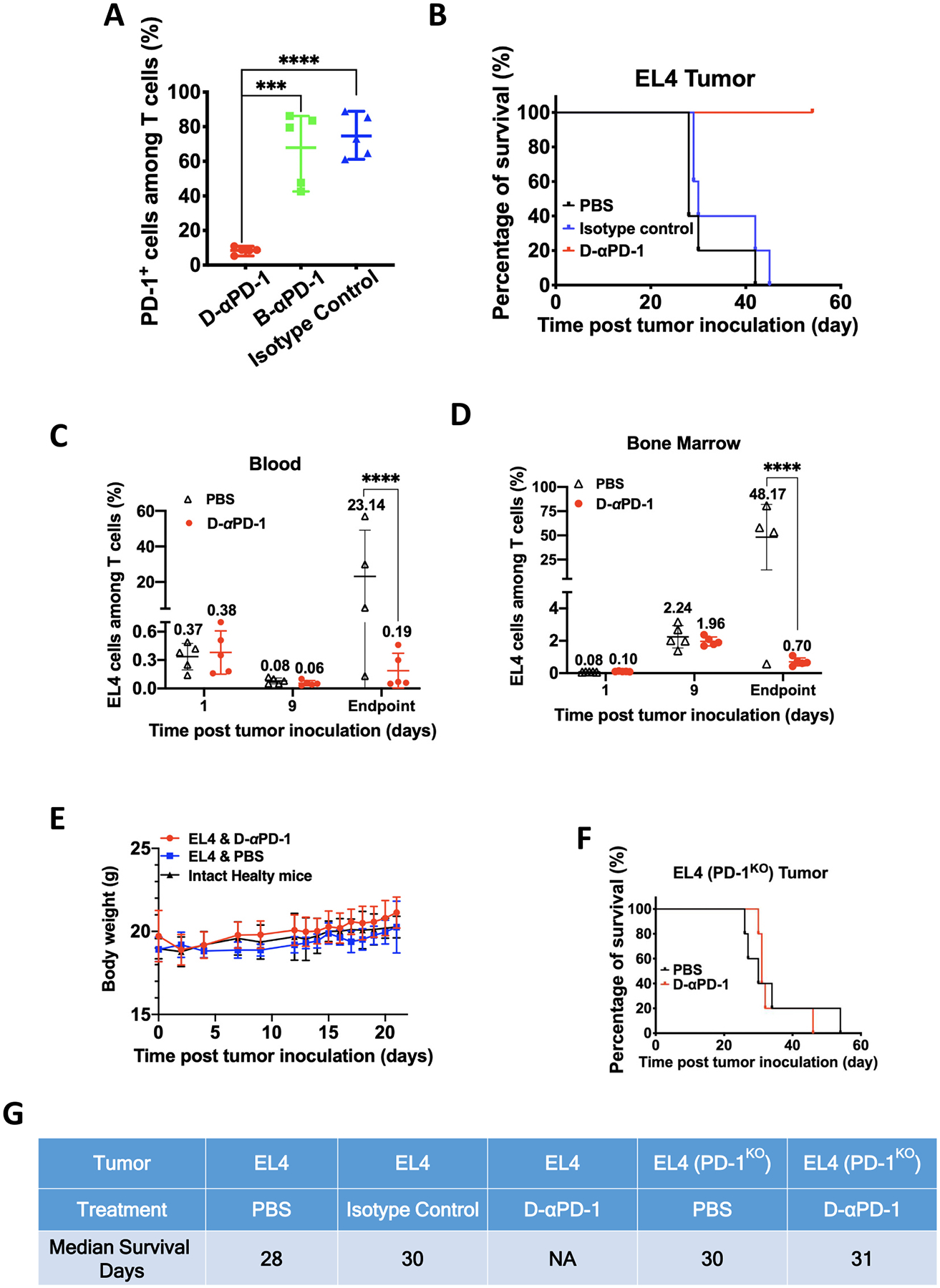
The *in vivo* depletion of PD-1^+^ cells by D-αPD-1. (A) Percentage of PD-1^+^ cells in T cells from bone marrow of mice which were inoculated EL4 cells and then treated with D-αPD-1, B-αPD-1, or IgG2a. The mice were sacrificed 10 days after inoculation. The data are presented as mean percentage ± SD of each treatment (*N* = 5, unpaired two-sided *t*-test). (B) Survival of C57/BL6 mice inoculated with EL4 cells followed by the i.p treatment of D-αPD-1, IgG2a or PBS. *N* = 5. (C) Percentage of EL4 cells in T cells from blood of mice which were inoculated EL4 cells and then treated with D-αPD-1 or PBS. Endpoint means the humane endpoint for mice in PBS treated group. D-αPD-1 treated mice were euthanized and examined at the time matching the endpoints of PBS-treated mice. Each symbol represents the EL4 fraction value of one mouse. The presented values are the means of EL4 cell fractions of treatment groups. (*N* = 4–5, unpaired two-sided t-test). (D) Percentage of EL4 cells in T cells from bone marrow of mice which were inoculated EL4 cells and then treated with D-αPD-1 or PBS. Endpoint means the humane endpoint for mice in PBS treated group. D-αPD-1 treated mice were euthanized and examined at the time matching the endpoints of PBS-treated mice. Each symbol represents the EL4 fraction value of one mouse. The presented values are the means of EL4 cell fractions of treatment groups. (*N* = 4–5, unpaired two-sided t-test). (E) Body weight change of mice with different treatments. N = 4–5. (F) Survival of C57/BL6 mice inoculated with PD-1 knock out EL4 cells followed by the i.p treatment of D-αPD-1 or PBS. *N* = 5. (G) Median survival times of mice with different treatments. (*P < 0.05; **P < 0.01; ***P < 0.001; ****P < 0.0001; ns: not significant).

Because the antibody-mediated cell depletion requires the involvement of a range of immune effector cells and molecules, it is difficult to reproduce complex conditions and assess antibody-mediated depletion *in vitro*. We therefore designed an *in vivo* depletion assay to assess the effectiveness of D-αPD-1 to deplete PD-1^+^ cells. Here, we first transferred EL4 cells into mice to boost numbers of PD-1^+^ cells in mouse bodies. Then, we treated mice with D-αPD-1, B-αPD-1, or the IgG2a isotype control at day 1 and day 10 after cell transfer. At 12 days after cell transfer, we quantified PD-1^+^ cells in bone marrows of transferred mice, where a large number of PD-1^+^ cells accumulated. We found the treatment of D-αPD-1 significantly reduced PD-1^+^ cells. Among D-αPD-1 treated mice, the mean fraction of PD-1^+^ cells among T cells is 8%. This mean is significantly lower than the means of B-αPD-1 and the isotype control-treated mice, at 68% and 75%, respectively (*P* < 0.001 and *P* < 0.0001) (Fig. 3A). This result indicates that D-αPD-1 has the capability to specifically eliminate PD-1^+^ cells *in vivo*. Compared to D-αPD-1, B-αPD-1 has the same antigen-binding sites for mouse PD-1 but lacks an IgG2a Fc. Thus, while B-αPD-1 can bind with mouse PD-1^+^ cells, it should not be able to initiate antibody-mediated elimination of these cells. On the other hand, compared to D-αPD-1, the isotype control has IgG2a Fc but not antigen-binding sites for mouse PD-1. Thus, the control should not eliminate PD-1^+^ cells. The above result confirmed these expectations.

EL4 is a tumor line syngeneic to C57B/L6 mice. EL4 cells inoculated into this strain of mice, if not eliminated, can grow into tumors that are lethal to the mice. Thus, we used survival of EL4-inoculated mice as an additional measurement of the depleting effect of D-αPD-1. Here, mice were inoculated with EL4 cells at day 0 and treated with D-αPD-1, IgG2a isotype control, or PBS at day 1. The median survival time for the PBS treated mice and the isotype control treated mice were 28 and 30 days after tumor inoculation, respectively (Fig. 3B and G). Further, no mice in these two treatment groups survived beyond 49 days. In contrast, all D-αPD-1 treated mice are still alive at 80 days after tumor inoculation, when this study was terminated (Fig. 3B and G). The survival of D-αPD-1 treated mice is significantly longer than PBS and the isotype control treated mice (*P* < 0.001). The 100% survival of the D-αPD-1 treated mice verifies the potent depleting effect of D-αPD-1 on PD-1^+^ cells. With the same design of EL4 cell transfer, we compared the presence of EL4 cells in mice that received PBS and D-αPD-1 treatment, respectively. We examine the cells at three time points, 1 and 9 days after cell transfer, and the humane endpoint for mice in the PBS treated group. Mice in the D-αPD-1 treated group were euthanized and examined at the time matching the endpoints of PBS-treated mice. We found EL4 cells are present at very low frequencies in blood and bone marrows of both treatment groups (Fig. 3C and D). However, at the endpoints, while D-αPD-1 treated group only have marginal amount of EL4 cells in blood and bone marrows, PBS treated mice have explosive numbers of EL4 cells. The average EL4 cell fractions among CD3 cells are 0.19% and 0.70% for blood and bone marrow samples of D-αPD-1 treated mice. These averages are significantly lower than those values of PBS-treated mice, 23.12% and 48.17% (*P* < 0.0001 for both comparisons). These results further reinforced that D-αPD-1 treatment eliminated EL4 cells in mice by targeting PD-1^+^ cells.

We also found the D-αPD-1 treatment was tolerated from the toxicity perspective, evidenced by body weight data of three treatment groups: intact mice, mice receiving EL4 cell transfer and the PBS treatment, and mice receiving EL4 cell transfer and the D-αPD-1 treatment (Fig. 3E). We measured body weights of these mice up to 21 days after the PBS and D-αPD-1 treatments. The mice received the D-αPD-1 treatment maintain the same body weight growth trend as the other two groups including intact healthy mice, indicating that the antibody does not cause any severe side effect that amounts to a body weight loss.

We further examined whether the depleting effect of D-αPD-1 depends on its binding with PD-1 on PD-1^+^ cells. For the examination, mice were inoculated with EL4 (PD-1^KO^) cells [15] and treated with either D-αPD-1 or PBS. In a sharp contrast to mice inoculated with EL4 cells, mice inoculated with EL4 (PD-1^KO^) cells did not respond to the treatment of D-αPD-1. The survival time of the D-αPD-1 treated group and the PBS treated group are not statistically different (*P* = 0.98, Fig. 3F). The mean survival days were 30 and 31 days post tumor inoculation, respectively (Fig. 3G). All mice in the D-αPD-1 treated group had to be euthanized by 6 weeks post tumor inoculation due to the growth of EL4 tumors. This result shows D-αPD-1 has no therapeutic effect on EL4 (PD-1^KO^) tumors, suggesting D-αPD-1 relies on PD-1 on PD-1^+^ cells to induce ablation of these cells.

### 2.4. D-αPD-1 depletes PD-1^+^ cells through CDC and ADCP

**Fig. 4.**
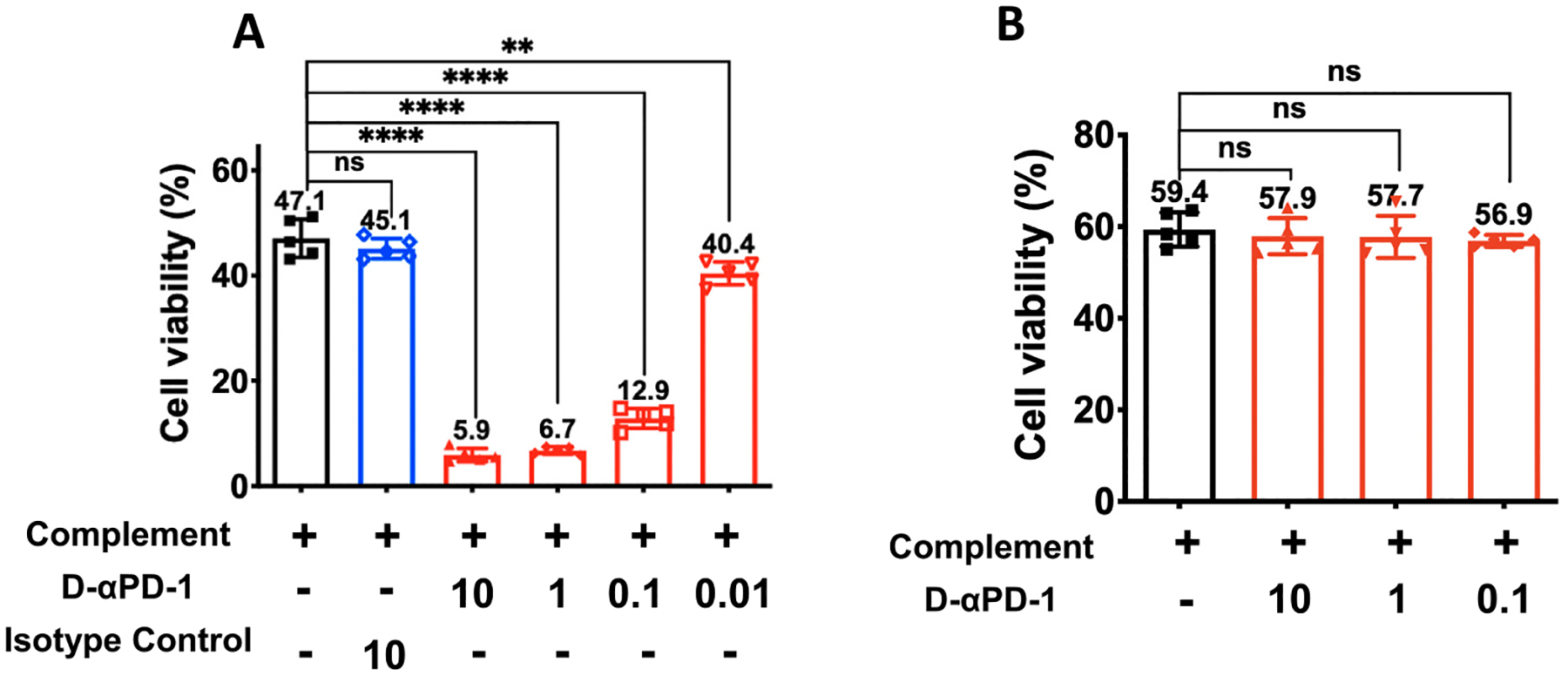
CDC of D-αPD-1. (A) Viability of EL4 cells after the cells were cocultured with baby rabbit complement and different concentrations (μg/mL) of D-αPD-1 or IgG2a for 3 h. (B) Viability of EL4 (PD-1^KO^) after the cells were cocultured with baby rabbit complement and different concentrations (μg/mL) of D-αPD-1 for 3 h. MTS assay was used to evaluate viability of the cells. Values represent the mean (±SD) percentage of live cells in all cells. N = 5. (**P < 0.01; ****P < 0.0001; ns: not significant).

We explored effector mechanisms that D-αPD-1 might utilize to deplete PD-1^+^ cells. The first examined mechanism was CDC, which is often employed by depleting antibodies. Here, target EL4 cells received six different treatments: complement only, complement plus D-αPD-1 at 4 different concentrations, and complement plus an IgG2a isotype control. The viability of EL4 cells after treatment were quantified and compared among treatments (Fig. 4A). The complement shows a baseline toxicity to EL4 cells, resulting in a viability of 47%. However, the addition of D-αPD-1 drastically enhance the toxicity to EL4 cells. The viability for EL4 cells that were treated with complement plus 10 μg/mL of D-αPD-1 decreased to one-eighth of the cells treated with the complement only (5.9% *vs* 47.1%, *P* < 0.0001). In contrast, EL4 cells treated with the complement plus 10 μg/mL isotype control showed the similar viability as the cells treated with the complement only (45.1% *vs* 47.1%, NS). These results suggest that D-αPD-1 is able to mediate CDC against PD-1^+^ cells. What further enhances this conclusion is the discovery that the effect of D-αPD-1 is concentration-dependent. When the concentration of D-αPD-1 used for the treatments increased from 0.01 μg/mL to 10 μg/mL, the viability of EL4 cells decreased from 40.4% to 5.9%. It is also noteworthy that all concentrations of D-αPD-1 in the range of 0.01 to 10 μg/mL caused significant reduction in viability as compared to the cells treated with the complement only, which highlights the potency of D-αPD-1 in mediating CDC. The above D-αPD-1-mediated CDC is dependent on PD-1 on EL4 cells. When EL4 (PD-1^KO^) cells were used to conduct the CDC assay, sharply different results emerged (Fig. 4B). The treatment of D-αPD-1 did not lower the cell viability compared with treatment of the complement only. Further, all three concentrations of D-αPD-1 yielded the same viabilities. These results together suggest that PD-1 on EL4 cells is required for the D-αPD-1-mediated CDC.

**Fig. 5.**
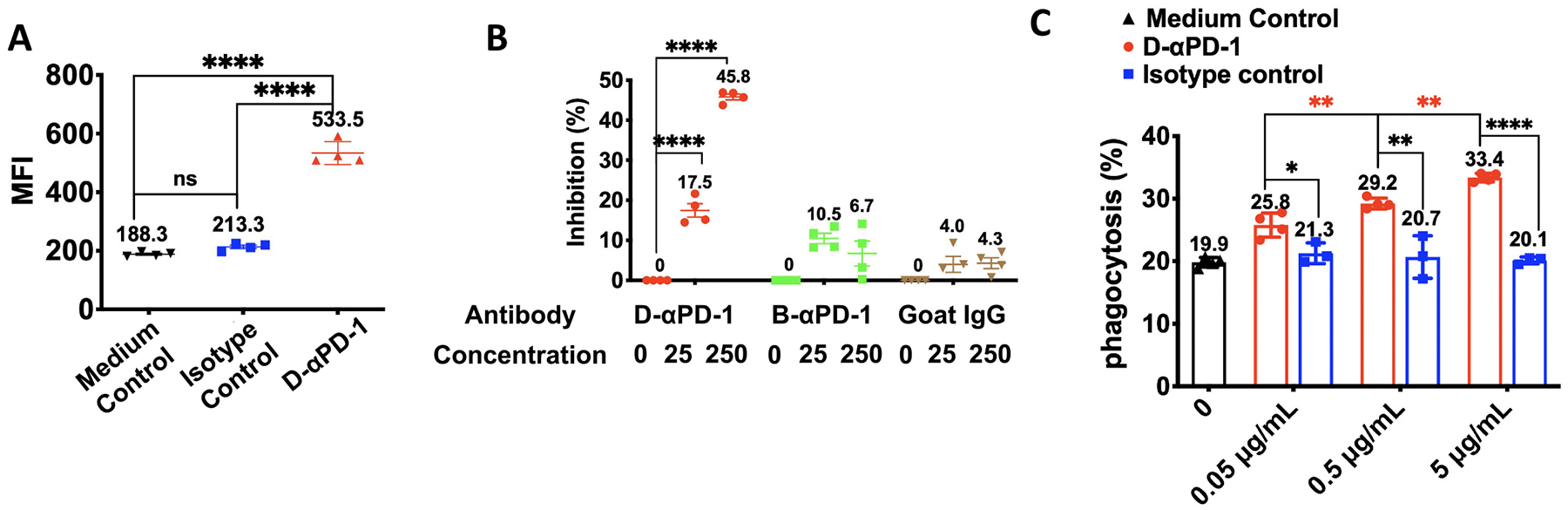
ADCP of D-αPD-1. (A) FcγRIV binding of Raw 264.7 cells to different Alexa-647-labeled-antibodies at 4 μg/mL at 4 °C for 30 min. The presented data are MFIs of cells after incubation ± SD (N = 4). (B) Inhibition effect of D-αPD-1 to the binding of anti-FcγRIV antibody with RAW 264.7 cells. The cells were treated with antibodies at different concentrations (μg/mL) at 4 °C for 30 mins before wash and stain with PE-anti-FcγRIV. The represented data are means of inhibition percentage ± SD (N = 5). (C) ADCP response towards PD-1+ EL4 cells by Raw 264.7 cell in the presence of D-αPD-1 or isotype IgG2a. The data are presented as the mean percentage of phagocytosis ± SD of each treatment (*N* = 3). (*P < 0.05; **P < 0.01; ****P < 0.0001).

Apart from CDC, ADCP was another mechanism we investigated. Before evaluating D-αPD-1-mediated ADCP by macrophages, we first assessed the binding between D-αPD-1 and macrophages since the binding is the prerequisite of ADCP. Here, RAW 264.7 cells were incubated with Alexa-647-labeled D-αPD-1, isotype control, or the medium control. Then, the bound antibodies were analyzed by flow cytometry (Fig. 5A). Compared to cells in the medium control, RAW 264.7 cells incubated with D-αPD-1 had an MFI as of 533.5, which was approximately 3 times higher (*P* < 0.0001). In contrast, isotype control treated cells had similar MFI as that of cells in the medium control (213.3 *vs* 188.3, NS). These results suggest D-αPD-1 binds with RAW 264.7 macrophages.

We next verified that the binding between D-αPD-1 and macrophages was mediated by mouse FcγRIV, a Fc receptor on macrophages that interacts with mouse IgG2a antibodies [33]. This study was completed through a competitive assay. Specifically, RAW 264.7 cells that are FcγRIV-positive were preincubated with D-αPD-1, B-αPD-1 (a rat IgG), and a goat IgG control. Then, all treated RAW 264.7 samples were stained with a PE-labeled anti-mouse FcγRIV antibody. The binding of D-αPD-1, B-αPD-1, and the goat IgG to FcγRIV were measure by their capacity to inhibit the association of anti-mouse FcγRIV antibody with RAW 264.7 cells (Fig. 5B). D-αPD-1 showed a salient inhibition capacity, reducing the binding of anti-mouse FcγRIV antibody by 17.5% and 45.8% when used at 25 μg/mL and 250 μg/mL, respectively (P < 0.0001). The treatment of B-αPD-1 and the goat IgG, on the other hand, only showed slight inhibition, approximately 10% each. And the inhibition resulted from B-αPD-1 and goat IgG were not concentration-dependent, which may be attributed to non-specific interactions between B-αPD-1 and goat IgG with RAW 264.7 cells. These inhibition data, together with the binding results above, not only confirmed D-αPD-1 bound with macrophages but also suggested that such binding was through FcγRIV.

Lastly, we examined ADCP using CFSE-labeled EL4 cells as the target cells and RAW 264.7 cells as the effector cells. The two cell populations were mixed at 1:1 ratio and incubated with increasing concentrations of D-αPD-1 and an IgG2a isotype control, or the medium control. Phagocytized target cells by RAW 264.7 cells were quantified using flow cytometry (Fig. 5C). At the all three antibody concentrations, D-αPD-1 resulted in a significantly higher percentage of phagocytosis as compared to the isotype control. For example, at the concentration of 5 μg/mL, the percentages of phagocytosis from the D-αPD-1 and isotype control treatments are 33.4% and 20.1%, respectively (*P* < 0.0001). The promotion of phagocytosis by D-αPD-1 is dose-dependent. When mixed cells were incubated with 0.05, 0.5 and 5 μg/mL D-αPD-1 antibody, the percentages of phagocytosis were 25.8%, 29.2%, and 33.4% respectively. In contrast, such a dose-dependent trend does not exist in the isotype control treated cell samples. Indeed, all isotype controls treated cell samples has the same low phagocytosis as that of the medium control. Together, these data pointed to the notion that D-αPD-1 can promote ADCP of PD-1^+^ cells.

## 3. Discussion

Here, we report the first depleting anti-PD-1 antibody, D-αPD-1. D-αPD-1 specifically binds with mouse PD-1^+^ cells and is able to utilize ADCP and CDC to phagocytose and ablate PD-1^+^ cells. D-αPD-1 induces the depletion of PD-1^+^ cells *in vivo*. The *in vivo* effect is strikingly potent since the treatment of D-αPD-1 effectively abolished PD-1^+^ target cells used in this experiment. These cells are transferred EL4 lymphoma cells and propagate robustly in mice if not eliminated. However, the treatment of D-αPD-1 is able to wipe out these transferred cells and keep transferred mice free from tumor growth and survive through the entire 80-day study.

A robust molecule or tool to deplete PD-1^+^ cells is desired for multiple reasons. First, a wide range of reports, including ours, suggest that PD-1^+^ lymphocytes play a critical role in the progression of autoimmune diseases [5,7,8,15]. Thus, depletion of these lymphocytes in autoimmune disease patients could be an appealing strategy to treat autoimmune diseases, as evidenced by our data in EAE and type-1 diabetes models [15]. Two additional advantages of this therapeutic strategy for autoimmune diseases are: first, the depletion covers both PD-1^+^ B cells and PD-1^+^ T cells so that it can abolish a wide and more comprehensive range of pathogenic immune cells in autoimmune diseases; second, the depletion only affects activated lymphocytes and keeps the vast majority of lymphocytes, which are naive lymphocytes, so that patients received the depletion treatment still possess the normal lymphocyte reservoir and are able to defend against future infections and malignancy.

Beside autoimmune diseases, the depletion of PD-1^+^ cells may also find utility in cancer immunological research and therapy. First, PD-1^+^ tumor cells have been found in a wide range of cancer types and are believed to contributed to the resistance to the PD-1 immune checkpoint therapy and hyper-progression associated with the therapy [21,40]. Thus, depletion of these PD-1^+^ tumor cells may offer a novel and much needed solution for the resistance and the hyper progression. Second, in tumors, PD-1^+^ effector T cells, PD-1^+^ Tregs, and PD-1^+^ tumor cells in some cases [19,24–28] create a PD-1 cell microenvironment. This environment is highly intertwined with the PD-1 immune checkpoint or the blockade of the checkpoint. The environment may dictate the outcome of the blockade therapy. On other hand, the blockade therapy itself may reshape the environment. Now, we have a depletion tool to profoundly change the environment and use the change to gauge the impact of the PD-1 cell microenvironment for tumor immunity and the success of PD-1 immune checkpoint therapy.

We have previously designed and developed an immunotoxin that depletes PD-1^+^ cells to treat autoimmune diseases [15]. However, we wonder if there is an alternative approach to deplete PD-1^+^ cells other than using an immunotoxin since immunotoxins cause safety concerns [41]. Different types of molecules, apart from immunotoxins, have been utilized for cell depletion, such as small molecules, antibodies, and antibody-drug-conjugates. Among these molecules, depleting antibodies are uniquely advantageous for their long plasma half-lives and plenteous preclinical and clinical information [42]. One clinically successful example is Rituximab, a depleting anti-CD20 antibody that is safely used to treat Non-Hodgkin’s Lymphoma (NHL), Chronic Lymphocytic Leukemia (CLL), and Rheumatoid arthritis (RA) [43]. Furthermore, two second generation depleting anti-CD20 antibodies ocrelizumab and ofatumumab are already approved for the treatment on multiple sclerosis and CLL, respectively. And another second generation depleting anti-CD20 antibody veltuzumab is currently under phase I/II clinical study [44]. Based on the accumulated knowledge of depleting antibodies, we decided to design and generate D-αPD-1. The C_H_ domains of D-αPD-1 is from mouse IgG2a, whose Fc fragment is able to bind with activating receptor FcγRIV with higher affinity compared with other antibody subtypes [33]. FcγRIV is strictly expressed on monocytes, macrophages, and neutrophils, which are the main effector cells that are able to deplete cells [33]. The variable domains (V_H_ and V_L_) of D-αPD-1 are designed based on B-αPD-1 [15], which makes it possible for D-αPD-1 to recognize and bind with mouse PD-1. The combination of these constant and variable domains enables D-αPD-1 to specifically bind to and eliminate mouse PD-1^+^ cells.

When PD-1^+^ cells were incubated with D-αPD-1 at 4 °C and 37 °C, cells at 37 °C accumulated more antibodies than cells at 4 °C. The greater accumulation may have two causes: greater number of antibodies bind with the cells at higher temperatures, or antibodies, together with antigens, are internalized by cells at a higher temperature. Whether these two causes are true or not is worth more investigations. On the other hand, we and others do have evidence that PD-1 is internalized [15,45]. For example, the PD-1 immunotoxin we designed needs to reach the cytoplasm of PD-1^+^ cells to exert its cytotoxicity, and our PD-1 immunotoxin has been found toxic to PD-1^+^ cells [15]. It is plausible that at least a fraction of D-αPD-1 that binds with PD-1 were internalized by PD-1^+^ cells. To further improve D-αPD-1, it is desirable to dampen internalization of the D-αPD-1/PD-1 complex. The functional design of D-αPD-1 requires it to stay on the cell surface for a sufficiently long time in order for effector cells and molecules to interact with the Fc of the antibody [46]. The internalization of depleting antibodies could be modulated by altering antibody structure [47] and using endocytosis inhibitors [48]. Interestingly, internalization was also identified as one reason for the resistance to Rituximab [49]. Yet, Obinutuzumab, a second generation of anti-CD20 antibody, is not sensitive to internalization, although the reason for such a difference is still not clear [50]. Overall, it is logical and practical to reduce the internalization of the D-αPD-1/PD-1 complex to boost the cell depletion effect of D-αPD-1.

In summary, D-αPD-1 specifically binds to and depletes PD-1^+^ cells. The depleting capacity makes the antibody a useful and reliable tool to investigate the role PD-1^+^ cells in both cancer and autoimmune diseases. The antibody could also become a treatment for patients with cancer or autoimmune diseases.

## 4. Materials and methods

### 4.1. Animals, cell lines and antibodies

Female C57BL/6 mice were purchased from The Jackson Laboratories. Animal studies were conducted following a protocol approved by the Institutional Animal Care and Use Committee (IACUC) at the University of Utah. EL4 (ATCC^®^ TIB-39^™^) cells were purchased from ATCC and were maintained in DMEM medium with 10% horse serum. EL4 (PD-1^KO^) cells were generated according to our previous research and maintained in the same medium for EL4 [15]. Macrophage Raw 264.7 cells were purchased from ATCC and maintained in RPMI 1640 medium with 10% FBS. Expi293 expression system was purchased from ThermoFisher and applied following the manufacture instruction. PE anti-mouse IgG2a antibody (clone: m2a-15F8) was purchased from Ebioscience. APC anti-mouse CD3 (clone: 17A2), FITC anti-mouse CD3 (clone:17A2), BV510 anti-mouse CD4 (clone: RM4–5), APC/Cyanine7 anti-mouse CD8 (clone: 53–6.7), PE anti-mouse TCR Vβ12 (clone: MR11–1), APC anti-mouse PD-1 (homemade), APC anti-mouse CD11b (clone: M1/70), PE anti-mouse PD-1 (clone: RMP1–14) and PE anti-mouse FcγRIV (clone: 9E9) were purchased from Biolegend.

### 4.2. Generation of expression vectors of D-αPD-1

The coding genes of the variable domains (V_H_ and V_L_) of D-αPD-1 are based on a rat anti-mouse PD-1 antibody RMP1.14, and constant domains are from mouse IgG2a (Genbank ID: BAC44883.1). Genes encoding light chain and heavy chain were separately inserted into pcDNA3.4 expression vector and synthesized by Thermofisher. The sequences of these two construct genes were verified by DNA sequencing (Genewiz).

### 4.3. DNA gel electrophoresis

1 μg heavy chain plasmid DNA was digested by 0.5 μL *Xba*I and 0.5 μL *Eco*RV, or 1 μg light chain plasmid DNA was digested by 0.5 μL *Sac*I and 0.5 μL EcoRV with 1 μL rCutSmart buffer in a 10 μL reaction at 37 °C for 3 h. Each sample with addition of 1 μL loading dye was loaded into the wells of a 1% agarose gel. The electrophoresis was run at 135 V for 30 min. The image of the gel was taken using the FluorChem FC2 imaging system (Alpha Innotech).

### 4.4. Protein expression and purification

B-αPD-1 was produced as previous report [51]. Heavy and light chains of D-αPD-1 encoded on two separate plasmids were cotransfected into Expi293 cells at ratio of 1:2 (heavy chain: light chain). The transfected cells were cultured at 37 °C with 5% CO_2_ for 6 days. Then the culture was collected and centrifuged. The supernatant was concentrated using Amicon ultra centrifugal filter units (30*K*). The antibody in the concentrated supernatant was purified through binding with Protein G beads (ThermoFisher). Antibody was eluted using 0.1 M Glyine (pH = 2.8) and neutralized with 1 M Tris-HCl (pH = 8.5). The yield, purity and integrity of the collected proteins were examined by both non-reducing and reducing SDS-PAGE analysis.

### 4.5. Evaluation of binding and uptake of D-αPD-1 by EL4 cells

For the evaluation of binding between D-αPD-1 and EL4 cells, 1 × 10^5^ EL4 cells were incubated with 0, 0.1, 1, and 10 μg/mL D-αPD-1 or control IgG2a at 4 °C for 30 min. EL4 cells were then stained with PE-anti-IgG2a and were analyzed by BD FACSCANTO II flow cytometer (BD Biosciences, San Jose, CA). For the evaluation of binding and uptake of D-αPD-1 by EL4 cells, D-αPD-1 was firstly labeled with Alexa Fluor 647 NHS Ester. Then 1 × 10^5^ EL4 cells were incubated with 0, 1, and 10 μg/mL labeled D-αPD-1 at 4 °C for binding only or 37 °C for binding and uptake for an hour. The EL4 cells were then analyzed using BD FACSCANTO II flow cytometer (BD Biosciences, San Jose, CA) and the mean fluorescence intensity (MFI) of the cells was reported.

### 4.6. In vivo EL4 cell depletion

3 × 10^6^ of EL4 cells were transferred into C57BL/6 mice through tail vein IV injection at day 0. The transferred mice were randomly separated into 3 groups and were respectively treated with 200 μg D-αPD-1, B-αPD-1 or IgG2a at day 1 and day 10. Mice were then sacrificed at day 12 and cells from bone marrow were collected from mice. Cells were then stained with APC anti-mouse CD3 and PE anti-mouse PD-1 and analyzed using BD FACSCANTO II flow cytometer (BD Biosciences, San Jose, CA). The fraction of PD-1+ cells among T cells (CD3+ cells) were quantified to show the EL4 cell depletion.

### 4.7. EL4 tumor inhibition and EL4 cell detection

2 × 10^4^ EL4 cells were transferred into C57BL/6 mice through tail vein IV injection at day 0. The transferred mice were randomly separated into 3 groups and were respectively treated with 200 μg D-αPD-1, IgG2a or PBS through IP injection at day 1. Their survival condition was observed through the whole study and median survival days were recorded.

2 × 10^4^ EL4 (PD-1^KO^) cells were transferred into C57BL/6 mice through tail vein IV injection at day 0. The transferred mice were randomly separated into 2 groups and were treated with 200 μg D-αPD-1 or PBS through IP injection at day 1. The median survival days of these two groups were recorded. 2 × 10^4^ EL4 cells were transferred into C57BL/6 mice through tail vein IV injection at day 0. The transferred mice were randomly separated into 2 groups and were respectively treated with 200 μg D-αPD-1 or PBS through IP injection at day 1. Mice were sacrificed at several datapoints, and cells from blood and bone marrow were collected from mice. Cells were then stained with FITC anti-mouse CD3, BV510 anti-mouse CD4, APC/Cy7 anti-mouse CD8, PE anti-mouse TCR Vβ12 and APC anti-mouse PD-1, and analyzed using BD FACSCANTO II flow cytometer (BD Biosciences, San Jose, CA). EL4 cells were gated using CD4-CD8-TCR Vβ12+ phenotype markers [52]. The fraction of EL4 cells among T cells (CD3+ cells) were quantified to show the EL4 tumor elimination.

### 4.8. In vitro ADCP assay

1:1 ratio of 1 × 10^5^ macrophage Raw 264.7 cells and 1 × 10^5^ CFSE-labeled EL4 cells were co-cultured with 0, 0.05, 0.5, and 5 μg/mL antibodies at 37 °C with 5% CO_2_ for 2 h. The cells were washed and stained with APC anti-mouse CD11b at 4 °C for 30 min. Then the washed cells were analyzed using BD FACSCANTO II flow cytometer (BD Biosciences, San Jose, CA) to evaluate the phagocytosis of EL4 cells by macrophage Raw 264.7 cells. EL4 cells were gated by FITC and SSC signals. Macrophage cells were gated by APC and SSC signals. FITC and APC double positive cells represent the EL4 cells engulfed by Raw264.7 cells. The percentage of phagocytosis was defined as: Number of FITC and APC double positive cells / Total number of Raw 264.7 cells.

### 4.9. Evaluation of D-αPD-1 binding with macrophage Raw 264.7 cells

D-αPD-1 or IgG2a were firstly labeled with Alexa Fluor 647 NHS Ester. Then 1 × 10^5^ macrophage Raw 264.7 cells were co-cultured with 4 μg/mL Alexa-647-labeled antibodies at 4 °C for 30 min. Cells were then washed and analyzed using BD FACSCANTO II flow cytometer (BD Biosciences, San Jose, CA) to evaluate the binding between D-αPD-1 and macrophage Raw 264.7 cells.

### 4.10. Binding inhibition study with D-αPD-1

1 × 10^5^ of macrophage Raw 264.7 cells were treated with D-αPD-1, B-αPD-1, and goat IgG at concentration of 0, 25, and 250 μg/mL at 4 °C for 30 min. Then the cells were stained with 2.5 μg/mL PE anti-mouse FcγRIV at 4 °C for 30 min. Cells were then analyzed using BD FACSCANTO II flow cytometer (BD Biosciences, San Jose, CA) to evaluate the binding between anti-FcγRIV and macrophage Raw 264.7 cells. Inhibition percentages of different groups were calculated based on the cells incubated without antibody treatment.

### 4.11. In vitro CDC assay

5 × 10^4^ EL4 cells per well were co-cultured with 1/30 baby rabbit complement dilution and D-αPD-1 at concentration of 0, 0.01, 0.1, 1, and 10 μg/mL, or 10 μg/mL IgG2a as control at 37 °C for 3 h. 5 × 10^4^ EL4 (PD-1^KO^) per well were co-cultured with 1/30 baby rabbit complement dilution and 0, 0.1, 1, and 10 μg/mL D-αPD-1 at 37 °C for 3 h. Then MTS (Promega) assay was then used for evaluation of EL4 cell viability as our previous research [15]. In brief, cells after incubation were incubated with MTS/PMS reagents for 4 h, then the OD_490_ of each treated sample was measured. The cell viability was calculated based on the equation:

$$\text{Cell viability}\left(\%\right)=\left({\text{OD}}_{\text{treated}}-{\text{OD}}_{\text{dead control}}\right)/\left({\text{OD}}_{\text{live control}}-{\text{OD}}_{\text{dead control}}\right)\times 100.$$

$${\text{OD}}_{\text{treated}}:{\text{OD}}_{490}\text{ of D-}\alpha \text{PD-}1\text{ or IgG}2\text{a treated cells}.$$

$${\text{OD}}_{\text{dead control}}:{\text{OD}}_{490}\text{ of dead control}\left(\text{Triton X-}100\text{ treated cells}\right).$$

$${\text{OD}}_{\text{live control}}:{\text{OD}}_{490}\text{ of the live control}.$$

### 4.12. Statistical test

Unpaired two-sided *t*-test were used to compare data other than survival data. The survival curves are calculated from different treatment groups using the Kaplan-Meier estimation approach and compared their difference using log-rank test. The level of test significance was defined as *p*-value <0.05. The survival analyses were conducted using statistical software R (version 4.1.2).
